# Development of Molecular Magnetic Resonance Imaging Tools for Longitudinal Tracking of Carotid Atherosclerotic Disease Using Fast Imaging with Steady-State Precession

**DOI:** 10.1007/s12975-022-01067-8

**Published:** 2022-07-18

**Authors:** Sung-Jin Park, Wan Ying Chan, Michael Ng, Yiu Cho Chung, Tze Tec Chong, Kishore Bhakoo, Joyce M. S. Chan

**Affiliations:** 1 Translational Cardiovascular Imaging Group, Institute of Bioengineering and Bioimaging (IBB), Agency for Science, Technology and Research (A*STAR), Singapore, Singapore; 2 Division of Oncologic Imaging, National Cancer Centre, Singapore, Singapore; 3 Siemens Healthcare Pte Ltd, Singapore, Singapore; 4 Department of Vascular Surgery, Singapore General Hospital, SingHealth, Singapore, Singapore; 5 Institute of Bioengineering and Bioimaging (IBB), Agency for Science, Technology and Research (A*STAR), Singapore, Singapore; 6 Lee Kong Chian School of Medicine, Nanyang Technological University, Singapore, Singapore

**Keywords:** Vulnerable carotid plaque, Stroke, Atherosclerosis, Fast imaging with steady-state precession (FISP), Iron, Magnetic resonance imaging (MRI), Inflammation

## Abstract

Identification of patients with high-risk asymptomatic carotid plaques remains a challenging but essential step in stroke prevention. Current selection criteria for intervention in carotid disease are still determined by symptomatology and degree of luminal stenosis. This strategy has been less effective in identifying the high-risk asymptomatic individual patients. Inflammation is the key factor that drives plaque instability causing clinical sequelae. Currently, there is no imaging tool in routine clinical practice to assess the inflammatory status within atherosclerotic plaques. Herein we describe the development of a novel molecular magnetic resonance imaging (MRI) strategy to interrogate plaque inflammation, and hence its vulnerability in vivo, using dual-targeted iron particle-based probes and fast imaging with steady-state precession (FISP) sequence, adding further prognostic information to luminal stenosis alone. A periarterial cuff was used to generate high-risk plaques at specific timepoints and location of the carotid artery in an apolipoprotein-E-deficient mouse model. Using this platform, we demonstrated that in vivo dual-targeted iron particles with enhanced FISP can (i) target and characterise high-risk vulnerable plaques and (ii) quantitatively report and track the inflammatory activity within carotid plaques longitudinally. This molecular imaging tool may permit (i) accurate monitoring of the risk of carotid plaques and (ii) timely identification of high-risk asymptomatic patients for prophylactic carotid intervention, achieving early stroke prevention.

## Introduction

Despite Level I evidence, the management of asymptomatic carotid atherosclerotic disease remains controversial. With advancement in medical therapies, yearly stroke risk has reduced to 0.5–1% [1]. Nonetheless, there are 10–15% asymptomatic patients who are likely to benefit from carotid intervention, as this clinical subgroup is still at higher risk for stroke even on best medical therapy [2]. The challenge is to identify these high-risk patients for prophylactic carotid intervention, leaving the majority of lower risk patients to be treated medically [1, 2]. Carotid magnetic resonance imaging (MRI) has vastly advanced the field of atherosclerosis imaging. The ability of in vivo carotid MRI to visualise and quantify the main hallmarks of plaque vulnerability has been extensively validated with histology [3]. In particular, carotid MRI detection of intraplaque haemorrhage and lipid-rich necrotic core are strong risk predictors for ischemic cerebrovascular events [3, 4]. Recently, molecular imaging strategies have been developed to look beyond anatomy by assessing the pathophysiological activities within plaques at the molecular level [5]. Molecular MRI has been used to directly report the inflammatory activities within the carotid plaques in mouse models [6–8]. Inflammation is not only instrumental in the development of atheromatous plaques, but, more importantly, plays a critical role in plaque destabilisation, converting chronic stable lesions into acute unstable ones with ensuing thromboembolism [9]. The increased expression of adhesion molecules, such as vascular cell adhesion molecule 1 (VCAM-1) and P-selectin (CD62P), on the activated endothelium promotes monocyte recruitment into the vascular tissues, thereby initiating and perpetuating the plaque inflammation [5, 9]. Herein, we have used fluorescent-labelled dual-targeted microparticles of iron oxide (DT-MPIO) against P-selectin and VCAM-1, the key inflammatory biomarkers in plaques. The synthesis and synergistic binding efficacy of DT-MPIO over single-ligand-MPIO have been reported previously [7, 8]. Using MR steady-state gradient echo sequence (GRE) developed specifically for molecular imaging of atherosclerosis, this strategy was utilised to (i) identify and characterise vulnerable plaques and (ii) quantitatively track inflammatory status of plaque progression, in the carotid arteries of an apolipoprotein E-deficient (ApoE^−/−^) cuff-implanted mouse model [7, 8, 10].

## Materials and Methods

### Synthesis of FITC-Tagged Dual-Targeted (DT) and Control IgG-MPIO

The synthesis of contrast agents dual-targeted (DT) and control IgG-MPIOs were reported previously [8]. Briefly, p-toluenesulfonyl surface functionalized MPIO (1 µm diameter; Dynabeads™ MyOne™ Tosylactivated; Invitrogen) were used to conjugate with VCAM-1 antibody (BD Pharmingen™), P-selectin antibody (CD62P; Santa Cruz Biotechnology) and fluorescein cadaverine (Life Technologies). Control immunoglobulin G IgG-MPIO was synthesised by conjugation with IgG-1 antibody (AbD Serotec) and fluorescein cadaverine.

### Animals and Cuff Implantation of Carotid Arteries

**Fig. 1:**
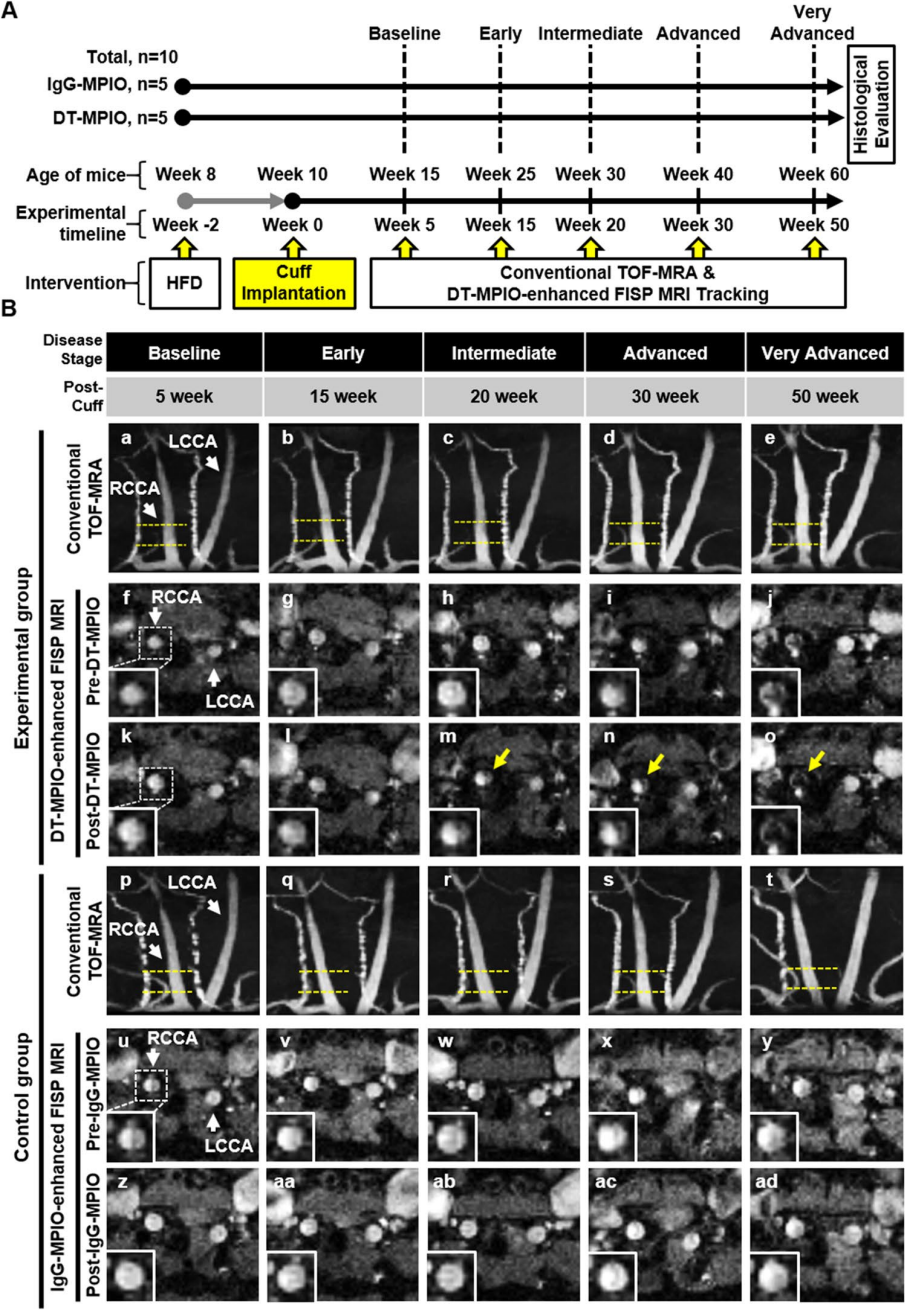
**A** Schematic diagram of experimental design. ApoE^−/−^ mice (DT-MPIO *n* = 5, IgG-MPIO *n* = 5) were used to develop the atherosclerosis model as published [10]. Briefly, mice commenced on a high fat diet (HFD) 2 weeks prior to surgical implantation of a shear-stress modifying cuff on RCCA, leaving LCCA untreated as internal control. HFD was maintained until end of study. Mice were imaged on MRI (11.7 T Bruker BioSpec) at the following timepoints post-cuff placement: 5 weeks (baseline), 15 weeks (early), 20 weeks (intermediate), 30 weeks (advanced) and 50 weeks (very advanced disease stage). At each MRI session, mice were first imaged with conventional TOF-MRA, followed by pre-contrast FISP, intravenous bolus of IgG/DT-MPIO and lastly post-contrast FISP. The region of interest (ROI) for FISP imaging was placed in the lower half of carotid arteries based on the high prevalence of high-risk plaques in that region as atherosclerosis progresses [10]. Histological analysis was performed at the end of last MRI session (50 weeks). **B** Longitudinal tracking of carotid plaques from early to advanced disease stages by in vivo MRI. (a–e) Conventional TOF-MRA shows minimal or no stenosis in lower part of RCCA (ROI was demarcated by the yellow dashed lines) from early (15 weeks) to very advanced disease stage (50 weeks) of the same animal. (f–o) DT-MPIO-enhanced MRA using FISP. In baseline (5 weeks) and early disease stage (15 weeks), no new distinct hypointense signal was detected in the same ROI of RCCA in the post-contrast FISP images. By contrast, as atherosclerosis progresses, in intermediate (20 weeks), advanced (30 weeks) and very advanced stage disease (50 weeks), new conspicuous hypointense signals were detected in the same ROI of RCCA post-DT-MPIO injection. (p–t) Conventional TOF-MRA shows minimal or no stenosis in lower part of RCCA (ROI was demarcated by the yellow dashed lines) from early (15 weeks) to very advanced disease stage (50 weeks) of the same animal. (u–ad) IgG-MPIO enhanced MRA using FISP. No new discrete hypointense signal was detected in post-contrast FISP images in the same ROI of RCCA at all stages of the disease

All animal experiments were approved by the Institutional Animal Care and Use Committee for Biological Resource Centre at A*STAR, Singapore (IACUC #191,459). ApoE^−/−^ mice (Taconic Biosciences) were used to develop the carotid atherosclerosis model by means of cuff implantation as previously described [7, 8, 10, 11]. The shear-stress modifying cuff induced development of vulnerable, inflamed plaques in the low shear-stress region upstream of cuff placement [7, 8, 10–12]. ApoE^−/−^ mice commenced on a high fat diet (HFD) 2 weeks prior to cuff placement. The cuff was surgically placed on the right common carotid artery (RCCA), while the left common carotid artery (LCCA) was left untreated as internal control. HFD was maintained until the end of study (Fig. 1A).

### In Vivo MRI of Carotid Arteries and Image Analysis

Mice underwent serial in vivo MRI (11.7 T Bruker BioSpec) to track the progression of atherosclerosis at the following timepoints post-cuff placement: 5 weeks (baseline), 15 weeks (early), 20 weeks (intermediate), 30 weeks (advanced) and 50 weeks (very advanced disease stage). At each MRI timepoint, mice were first imaged with conventional 3-dimensional time-of-flight (TOF) MR angiography (MRA) to assess degree of luminal stenosis for plaque progression from early to advanced disease stage [8]. A region of interest (ROI) was selected in the carotid arteries for axial imaging using steady-state GRE sequences. The ROI was placed in the lower half of carotid arteries based on the high prevalence of high-risk vulnerable plaques developed in that region as atherosclerosis progresses, validated in our previous work [10]. Once identified, baseline pre-contrast fast imaging with steady-state precession (FISP) sequence was performed: TR, 9.2 ms; TE, 4.6 ms; FOV, 25 × 25 mm; acquisition matrix, 256 × 256; flip angle, 15; averages, 30; slices, 3; slice thickness, 0.3 mm; and acquisition time, 3 m 40 s. After pre-contrast baseline scans, a bolus of DT-MPIO (*n* = 5) or control IgG-MPIO (*n* = 5) contrast agent, 30 mg iron/kg body weight, was injected intravenously, while the animal still positioned in the scanner. Thereafter, post-contrast FISP sequences were repeated for 2 h (Fig. 1A). MR images were analysed using ImageJ by delineation of RCCA of FISP images as ROI. Percentage difference between pre- and post-contrast signal-to-noise ratio (SNR) in FISP images was calculated:

$$\frac{SNRpost-SNRpre}{SNRpre } \times 100\%$$

where $${\mathrm{SNR}}_{\mathrm{ROI}}=\frac{mean\  intensity\  ROI}{mean\  intensity\  background\  noise}$$

The SNR difference (%) between pre- and post-contrast FISP images in both DT-MPIO group and IgG-MPIO group were calculated.

### Histology

At the end of the last MRI timepoint (50 weeks), carotid arteries were harvested.

Histology, immunohistochemistry staining and analysis were performed in matching regions of carotid arteries as described [8, 10].

## Results

**Fig. 2:**
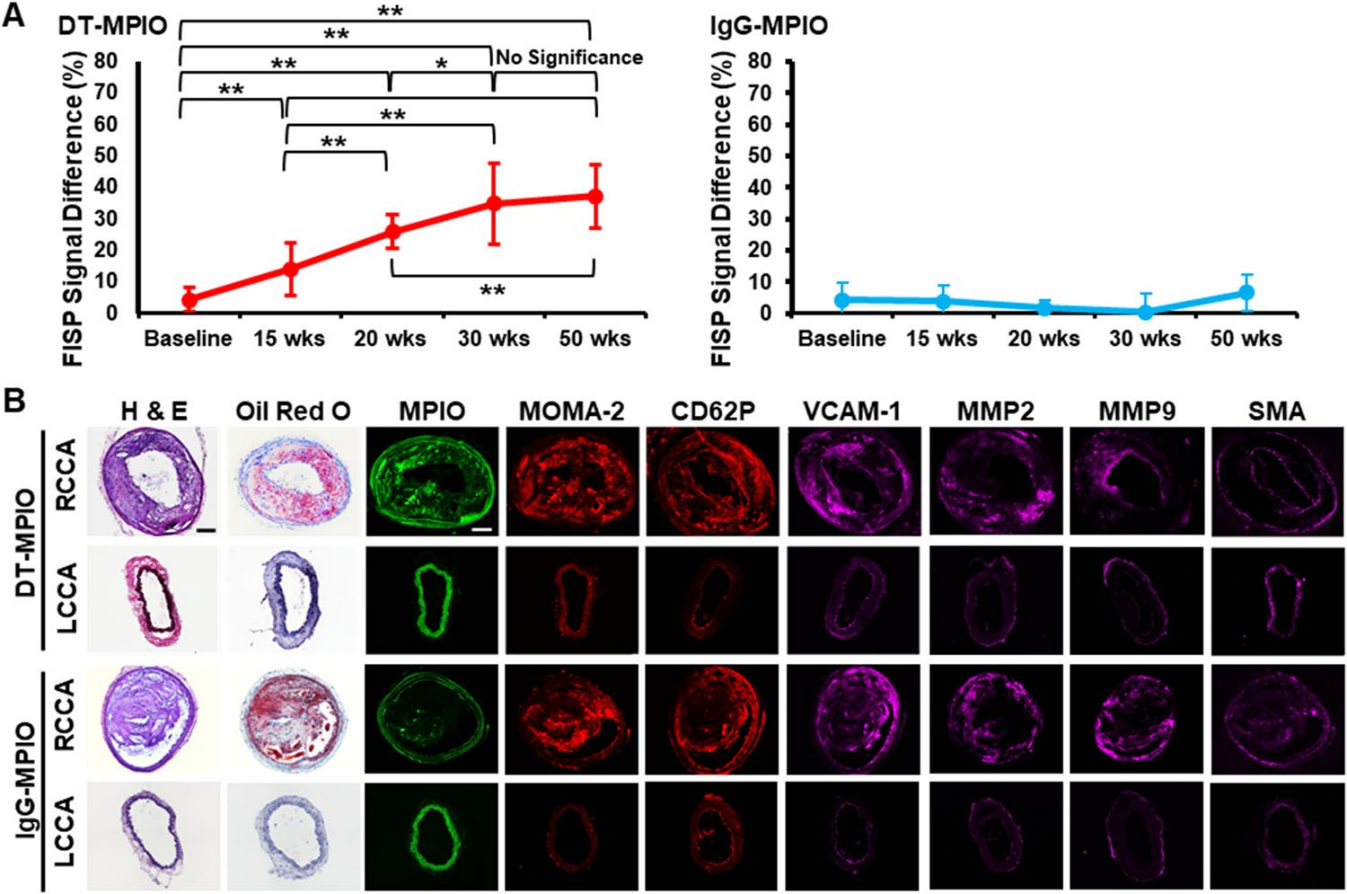
**A** Magnitude of DT-MPIO-induced hypointense signal increased as atherosclerosis progressed in cuff-implanted RCCA, while IgG-MPIO-induced signal remains low throughout. Percentage difference between pre- and post-contrast SNR in FISP images (DT-MPIO *n* = 5, IgG-MPIO *n* = 5) were calculated for statistical analysis using Student’s *t*-test. *p < 0.05, **p < 0.01. **B** Histology of matching regions in RCCA and LCCA in both DT-MPIO and control IgG-MPIO groups. Histological analysis was performed in matching regions of carotid arteries (demarcated by yellow dashed lines) after imaging at week 50 post-cuff placement as described [8]. Histology confirmed that ample amount of fluorescent-labelled DT-MPIO was identified within the intima of the plaques in matching region of RCCA. These plaques bear vulnerable inflamed plaque phenotype (i.e. high expression of inflammatory biomarkers: MOMA-2, CD62P (P-selectin), VCAM-1, MMP2, MMP9, high “destabilising” lipid content, thin layers of “stabilising” smooth muscle cells in the cap of plaque). Histology sections in the IgG-MPIO group showed similar vulnerable inflamed plaque phenotype but with minimal amount of fluorescent-labelled IgG-MPIO identified within the intima of the plaques in matching region of RCCA. Green autofluorescence of elastic lamina was observed in the outermost part of tunica intima of both RCCA and LCCA as seen in previous study [8]

TOF-MRA demonstrates minimal or no stenosis in lower part of RCCA from early to very advanced disease stage (Fig. 1B a–e). DT-MPIO-enhanced MRA using FISP was performed after TOF-MRA (Fig. 1Bf–o f–o). In baseline and early stage, no new distinct hypointense signal was detected in lower part of RCCA. In intermediate, advanced and very advanced stage, new conspicuous hypointense signal was detected in the lower part of RCCA post-DT-MPIO injection. Conversely, from the IgG-MPIO post-contrast FISP images (Fig. 1B z-ad), no new hypointense signal was detected in all disease stages. Furthermore, the magnitude of change in hypointense signal, induced by DT-MPIO, was greater as atherosclerosis progressed, with the greatest signal changes in advanced stages, while the magnitude of signal change in the IgG-MPIO group remained low throughout all disease stages (Fig. 2A).

Histology confirmed that significant amount of fluorescent-tagged DT-MPIO was identified in plaques in matching region of RCCA. These plaques bear vulnerable inflamed plaque phenotype (i.e. high expression of inflammatory biomarkers: MOMA-2, P-selectin, VCAM-1, MMP2, MMP9, high “destabilising” lipid content, thin layers of “stabilising” smooth muscle cells in the cap of plaque) (Fig. 2B). By contrast, the control group presented only minimal amount of non-specific IgG-MPIO binding to vulnerable inflamed plaques. The histology corroborated that (i) the new conspicuous hypointense signal on the post-contrast FISP images in DT-MPIO group (Fig. 1B m–o) was attributed to DT-MPIO targeting at the high-risk plaques (Fig. 2B) and (ii) the absence of new discrete hypointense signal on the post-contrast FISP images in IgG-MPIO group (Fig. 1B z–ad) were consistent with the lack of IgG-MPIO binding in high-risk plaques (Fig. 2B).

## Discussion

Current selection criteria for surgical intervention for carotid atherosclerosis are still dependent on symptom status and luminal stenosis, evaluated by conventional angiographic tools. The Asymptomatic Carotid Surgery Trial (ACST), however, highlighted a subgroup of high-risk asymptomatic patients bearing vulnerable plaques, which traditional angiographic methods failed to detect [13]. To overcome this shortfall, promising non-invasive imaging tools have been developed to interrogate plaque inflammation and vulnerability. ^18^F-FDG PET imaging and contrast-enhanced ultrasound may provide useful information for risk stratification of carotid atherosclerotic disease and prediction of early stroke recurrence [14–16]. Carotid MRI enables identification and quantification of the high-risk plaque components, such as surface disruption, intraplaque haemorrhage and lipid-rich necrotic core, which are associated with future cerebrovascular events [17, 18]. Despite significant advancement in plaque MRI techniques, direct reporting of the underlying inflammatory activities in local plaques was hampered until the utilisation of USPIO [19, 20] and MPIO [7, 21, 22] in molecular MRI.

Herein, we have developed a novel adjunctive imaging strategy to investigate plaque inflammation and vulnerability in vivo, providing prognostic information beyond luminal stenosis. To our knowledge, this is the first pre-clinical study to develop a molecular MR imaging tool using DT-MPIO probes together with FISP sequence to identify, characterise and longitudinally track plaque inflammation and vulnerability. FISP capitalises on the sensitivity of T2* weighting to iron of gradient echo sequence, resulting in higher SNR per unit scan time, yielding superior strategy over the existing methods. Using the same echo time, FISP provides T2 weighting and higher SNR without compromising T2* sensitivity when compared to conventional T2* spoiled gradient echo and TOF imaging [23]. T1 weighted imaging sequences have been used for identifying features of high-risk plaques. For instance, methaemoglobin in haemorrhage is best visualised by magnetisation-prepared rapid acquisition with gradient echo (MPRAGE) [24]. Lipid core can be detected by comparing pre- and post-contrast T1-weighted sequences [25]. Exploiting FISP’s T2* sensitivity to iron warrants augmentation of DT-MPIO-induced signal effect to target specific inflammatory activity within vulnerable plaques. This susceptibility-induced contrast enables visualisation of the underlying pathophysiological process within the plaques, providing additional information beyond T1-weighted imaging of plaque composition. This molecular imaging strategy can achieve conspicuous and quantifiable image contrast in vivo, making it valuable for monitoring the risk of carotid plaques.

In this study, the detection of only minimal/low-grade luminal stenosis by TOF-MRA, even in advanced disease, highlights one of the important limitations of conventional angiographic techniques: vulnerable plaques could be missed due to expansive vascular remodelling. It is generally accepted that luminal stenosis alone is inadequate to reflect the true disease burden due to positive arterial remodelling [26]. Remodelling may present normal luminal angiograms despite the presence of a large vulnerable plaque which may be at risk of rupture and subsequent embolization [27, 28]. Supporting this, neither ACST [13] nor Asymptomatic Carotid Atherosclerosis Study [29] trials showed any evidence that stenosis severity was predictive of a higher risk of late stroke. Management of carotid atherosclerotic disease relying on degree of luminal stenosis alone resulted in a mere 7% reduction in stroke [30]. To date, stenosis severity is ineffective in identifying a high-risk asymptomatic subgroup [31].

Using DT-MPIO-enhanced FISP, however, plaque inflammation and vulnerability were characterised and quantitatively tracked longitudinally, proportionate to the degree of hypointensity signals as atherosclerosis progressed. This concurred with our progressive atherosclerosis model, in which plaques formed in lower part of RCCA were high-risk plaques (American Heart Association Type V, VI) [32, 33], with higher level of inflammation and vulnerability index, from intermediate (20 weeks) to very advanced disease stage (50 weeks post-cuff placement) [10]. This molecular imaging tool may overcome current limitations of conventional angiography to look beyond lumen stenosis by directly reporting inflammatory activity within plaques. The molecular imaging tool can potentially target fewer, but high-risk asymptomatic, patients for prophylactic carotid intervention, sparing majority of lower risk patients from procedures with risk of perioperative stroke and of limited utility [1]. The direct reporting of intraplaque inflammation may add substantial assurance not only to dose selection, but also decision-making for embarking on large-scale clinical endpoint trials, saving significant costs and ultimately accelerating the availability of novel cardiovascular therapeutics.

## Data Availability

The data that support the findings of this study are available from the corresponding author upon reasonable request.
